# Therapeutic Adherence and Functional Capacity in Heart Failure

**DOI:** 10.5935/abc.20150114

**Published:** 2015-09

**Authors:** Márcia Maria Carneiro Oliveira, Elieusa e Silva Sampaio, Roque Aras Júnior

**Affiliations:** Universidade Federal da Bahia – UFBA, Salvador, BA – Brazil

**Keywords:** Heart Failure, Medication Adherence, Patient Care

**Dear Editor,**

Heart failure (HF) has become a major public health problem, as it is the final pathway of most heart diseases^1^. One of the main factors that lead to decompensation in HF is poor patient adherence to treatment^2^.

Educational intervention programs for the management of chronic disease and clinical monitoring of HF are associated with better adherence to treatment^3,4^. It was observed, in a non-controlled clinical trial with 25 patients in a HF outpatient clinic, that the educational intervention improved the following indicators: renal function assessment with improved estimated glomerular filtration rate (eGFR; median: V0 = 61 vs. V1 = 68) and B-type natriuretic peptide (BNP), treatment adherence score and functional capacity at the six-minute walk test (6MWT). The treatment adherence questionnaire assessed the following: correct use of medications, daily weight, salt and fluid restriction, alcohol intake and attendance at appointments and tests. The adherence score can range from zero to 10 points^4^. At the adherence assessment, an improvement was observed comparing the pre (V0) and post (V1) intervention periods, with a median of V0 = 5.0 *vs*. V1 = 6.1 (p = 0.006). It was demonstrated that in V0, the patients considered non-adherent sought the emergency service in the last 30 days more often than those considered adherent (p = 0.013) and in V1, 100% of patients reported not having sought the emergency service during the period. After the educational intervention, patients with systolic HF with low left ventricular ejection fraction (LVEF ≤ 40%) showed improved adherence score (p = 0.006) and clinical improvement with a decrease in functional class (FC) and weight (p = 0.022). As for the 6MWT, the patients that showed better performance in the test were those considered adherent to treatment, with FC III, time of HF = 12-16 years, LVEF of 24 to 29%, and hypertensive etiology. Clinical and renal function improvement was observed in outpatients with heart failure submitted to educational intervention that showed greater adherence to optimized therapy.

## References

[1] Reis FJ, Fernandes AM, Bahia RL, Sahade V, Rodrigues Junior ES (2007). A importância de serviços especializados e multidisciplinares para
pacientes com insuficiência cardíaca e seu impacto na saúde
pública. Gazeta méd Bahia.

[2] Fonarow GC, Abraham WT, Albert NM, Stough WG, Gheorghiade M, Greenberg BH, OPTIMIZE-HF Investigators and Hospitals (2008). Factors identified as precipitating hospital admissions for heart
failure and clinical outcomes: findings from OPTIMIZE-HF. Arch Intern Med.

[3] Cruz FD, Issa VS, Ayub-Ferreira SM, Chizzola PR, Souza GE, Moreira LF (2010). Effect of a sequential education and monitoring programme on
quality-of-life components in heart failure. Eur J Heart Fail.

[4] Bocchi EA, Cruz F, Guimarães G, Pinho Moreira LF, Issa VS, Ayub Ferreira SM (2008). Long-term prospective, randomized, controlled study using repetitive
education at six-month intervals and monitoring for adherence in heart failure
outpatients: the REMADHE trial. Circ Heart Fail.

